# Robotic 3D scanner as an alternative to standard modalities of medical imaging

**DOI:** 10.1186/2193-1801-3-13

**Published:** 2014-01-06

**Authors:** Adam Chromy, Ludek Zalud

**Affiliations:** Central European Institute of Technology, Brno University of Technology, Technicka 10, 616 00 Brno, Czech Republic; Faculty of Electrical Engineering and Communication, Brno University of Technology, Technicka 12, 616 00 Brno, Czech Republic; International Clinical Research Center - Center of Biomedical Engineering, St. Anne’s University Hospital Brno, Brno, Czech Republic

**Keywords:** 3D model of human body, 3D scanner, Medical imaging, MRI alternative

## Abstract

There are special medical cases, where standard medical imaging modalities are able to offer sufficient results, but not in the optimal way. It means, that desired results are produced with unnecessarily high expenses, with redundant informations or with needless demands on patient. This paper deals with one special case, where information useful for examination is the body surface only, inner sight into the body is needless. New specialized medical imaging device is developed for this situation.

In the Introduction section, analysis of presently used medical imaging modalities is presented, which declares, that no available imaging device is best fitting for mentioned purposes. In the next section, development of the new specialized medical imaging device is presented, and its principles and functions are described. Then, the parameters of new device are compared with present ones. It brings significant advantages comparing to present imaging systems.

## Introduction

In order to understand all the processes inside the human body, or in order to detect its defects, we want to be able to look into the body in non-invasive way, without its damaging – and that is what we achieve using medical imaging. This important tool has ever played an important role in public health care since the first medical imaging technique was presented at 1985 by Wilhelm Rontgen (Novelline 1997) and has been developed a lot since this time. Nowadays, we have plenty of devices using different techniques, when most of them are focused on seeing the interior objects inside the body and some of them are able to build 3D model of part of human body (Singh and Jones 1993).

But there are many specific cases, where information about inner structures is useless for our purpose and just 3D model of surface is sufficient. As an representative example, monitoring of rehabilitation process after serious injury can be mentioned: The monitored value is volume of musculature and the shape of recovering muscle. The gain in muscle volume is too small to be measureable by common techniques and changes in shape are also tiny. As a result of this, the only solution is to compare detailed 3D models of damaged part of patients body acquired in different time moments (Konecny et al. 2013). Because the monitored values can be measured from body surface, the required model for this case is a 3D model of surface, inner structures are not necessary.

Actually, common imaging modalities capable of building 3D models of surface are Computed Tomography (CT), Magnetic Resonance Imaging (MRI) and partially Ultrasonography (Udupa and Herman 2000: Webb 2002). Each imaging technique has its advantages and disadvantages:

### Ultrasonography

Medical ultrasonography is a modality, which is primarily intended to watch internal constitution of inner tissues, not to capture the body surface (Robertson and Baker 2001). Although ultrasonography in a broader sense, on account of its physical principle, is capable of capturing surface, this technique is not sufficient because of its poor resolution, which is caused by sound-beam divergence (Musil et al. 2008). In order to analyze volume and tissue shape, resolution higher than provided by ultrasound is required.

### Computed tomography

Computed Tomography reaches up to 0.2 mm spatial resolution in output 3D model (MCCollough and Zink 1999), what is value, which is fully sufficient for our purposes. It works on principle, that high number of 2D scans is produced using X-ray source and these slices are merged together into one 3D model (Herman and Gabor 2010). Ionizing radiation absorbed by patient during one scan is up to 15 mSv, what is one third of allowed exposition for workers with ionizing source per year and for common people exceeds allowed hygiene limits even 15 times (Statni urad pro jadernou bezpecnost 2002). For this reason, use of this modality is allowed as rare as possible and repeated scanning is completely out of the question.

### Magnetic resonance imaging

Compared to technologies mentioned above, Magnetic Resonance Imaging is the best fitting modality for purposes of capturing 3D models of surface periodically. It doesn’t work with X-rays, so there is no limitation on number of models scanned. Its resolution is also high enough (approximately 1 mm) (Novelline 1997) and this technique produces 3D model containing both surface and inner structures (Seidl and Vaneckova 2007). But because of its complexity, acquisition costs of MRI scanner begins at 750 000 EUR, and price per one model reaches from 230 to 350 EUR (Magnetic Resonance Imaging 2010).

### Summary

As summarized above, none of these technologies are intended to provide 3D model of patient’s body surface only. All of them provides image of inner structures, what influences device’s costs and also brings another disadvantages. There is no imaging device intended specially for medical cases, which requires model of body surface as a tool for examination.

To develop such specialized device, which will be free of disadvantages mentioned above, it is aim of project described in this paper. In the following section, the constitution and main operating principles of this new proposed medical imaging device are described, then comparison with present devices is made.

## Robotic 3D scanner

**Figure 1:**
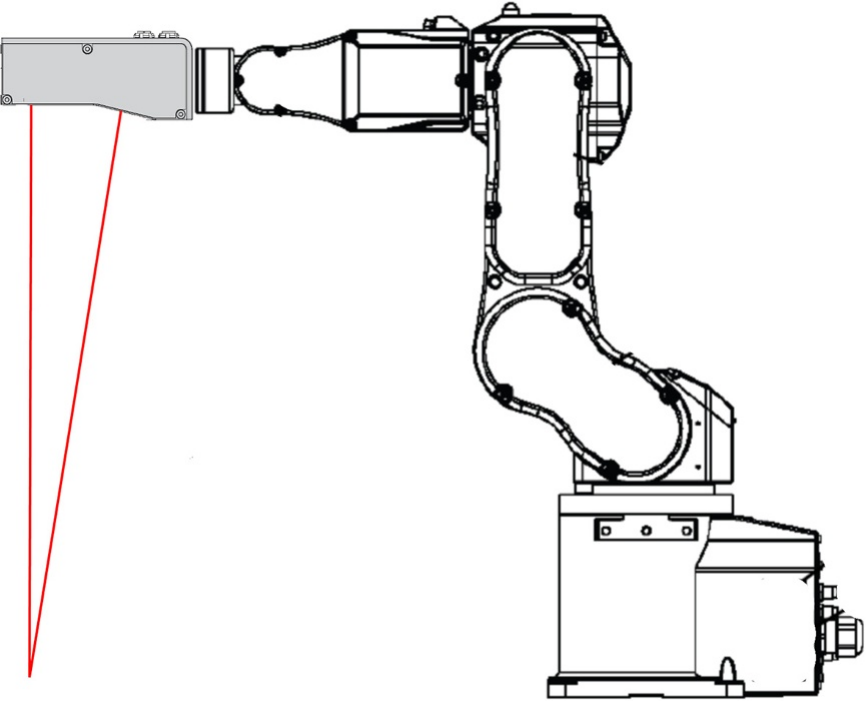
**Robotic 3D Scanner overview.** Laser scanner is mounted directly on the manipulator’s end-point and can be easily unmounted by unscrewing four screws and unplugging Ethernet cable and power plug. It allows easy change of used laser scanner in dependence on object size.

Proposed Robotic 3D Scanner consists of laser scanner, which is mounted on industrial manipulator’s end-point (Figure 1) and can be easily replaced with any other. Algorithms and control software are fully independent on type of scanner used, the only requirement is, that scanner’s driver must implement defined interface. This provides significant flexibility – we are able to scan wide range of objects.

In case of scanning surface of human body, high resolution is required, so we use precise laser scanner based on triangulation principle. These scanners are very accurate, but disposes with small measuring range. Such scanner should also use laser emitter compatible with Class 1 or Class 1M in order to avoid damage of patient eyes in case of scanning his face, what is realizable with some scanners presently available.

If we want to make 3D model of any large structure, like a building etc., it is also possible, we can only change the scanner to one with wider measuring range, for example laser scanner based on TOF principle can be used (Shan and Toth 2008). So this device is not just for scanning body – it is flexible multi-purpose 3D scanner.

### Physical constitution

**Figure 2:**
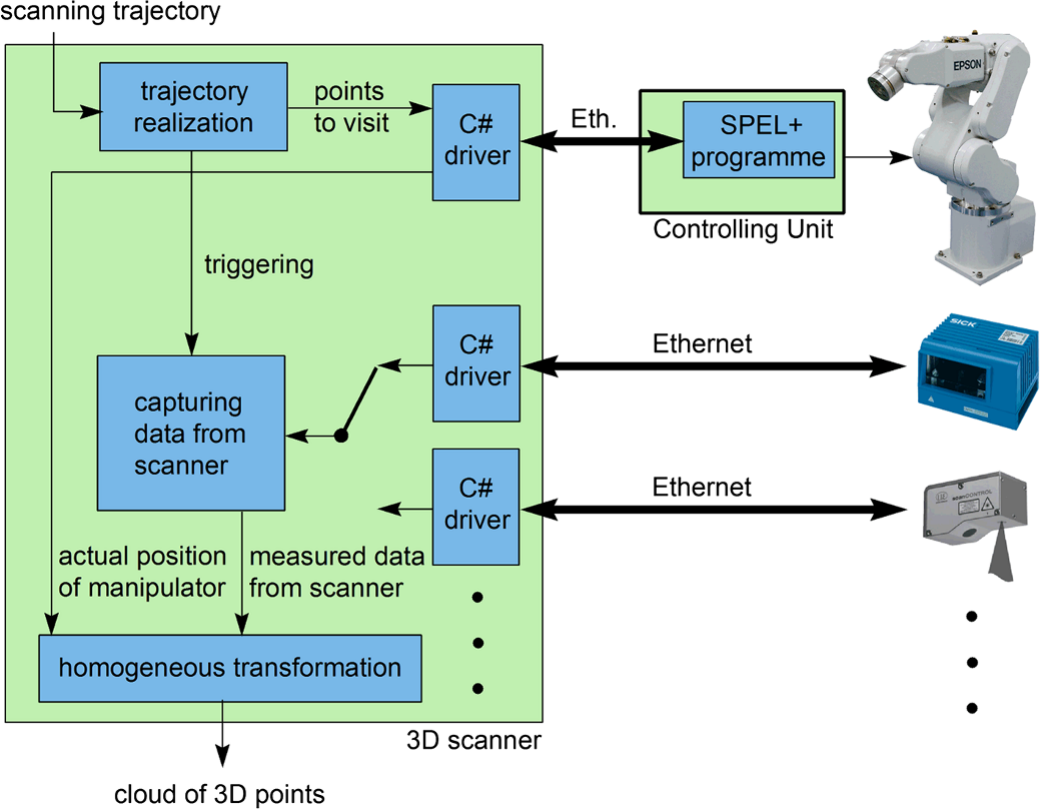
**Block scheme of essential parts of Robotic 3D Scanner.** Desired scanning trajectory enters the system in the from of list of points to measure from, these points are consequently visited, data are measured and point-cloud in 3D space is output to be further processed.

Block scheme of essential parts of the Robotic 3D Scanner is displayed on Figure 2.

Both devices are connected to the controlling computer using Ethernet. At this computer, there is a software acquiring measured data from scanner and information about actual position from robotic end-point. These data are then processed to form of point-cloud using Homogeneous transformation.

Robotic manipulator’s end-point is moved from point to point over the predefined scanning trajectory and when desired point is reached, the measuring is performed and data are processed. Each positioning command is validated by driver when desired position is reached, what ensures, that scanning is performed from exact point of view. Communication between manipulator and control computer is performed via driver communicating with program in the control unit of robot, which receives commands sent over Ethernet and moves with manipulator (Chromy 2013). This empowers real-time control of robotic manipulator. Scanning trajectory is defined as a list of points to measure from and can be easily defined and managed using graphical interface.

### Measured point coordinates computation

Each 3D point is computed using data from laser scanner, information about actual position of manipulator’s end-point and time-invariant parameters describing mounting of scanner at the manipulator’s end-point. All these acquired data are processed using Homogeneous transformation approach. Data from laser scanner are transformed from its own coordinate system *S* into our coordinate system 0 through coordinate systems *M* and *E*. This transformation *H*_0*S*_ could be defined as a sequence of essential transformations among neighbour coordinate systems (Chromy 2013):
1

**Figure 3:**
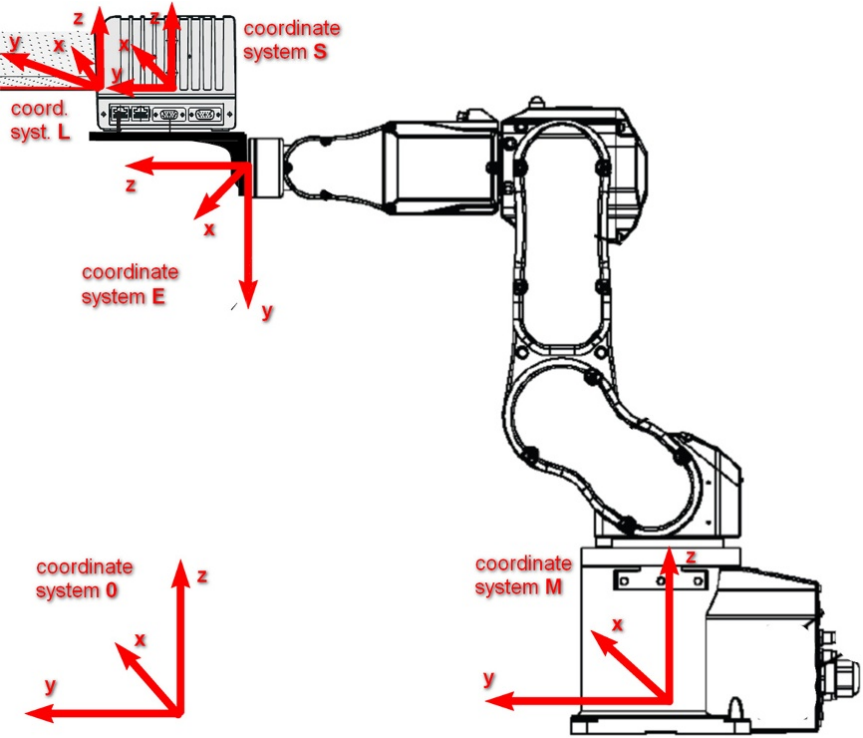
**Overview of used coordinate systems.** S – internal coordinate system of laser scanner. In this coordinate system, measured profile is output from scanner driver. Some scanners produce data in the form of distance (L) and angle. In this case, additional transformation from L to S is performed inside scanner’s driver. E – coordinate system with origin at manipulator’s end-point. M – internal coordinate system of manipulator. 0 – default coordinate system into which data are transformed.

All used coordinate systems are shown at Figure 3 and essential transformations among neighbour coordinate systems are described in the following text.

### Transformation L → S

Describes relation between coordinate system of laser range finder inside laser scanner and laser scanner itself and must be performed just in case, when laser scanner provides profile data in the form of measured distances and actual rotations of sweeping mechanism *α* instead of set of points in its own 3D coordinate system *S*. This preprocession is defined as rotation along *Z* axis by angle *α*:
2

If data from laser scanner are produced in Cartesian coordinate system form, this transformation is not performed. On output of this transformation, linear block of points (2D line) is placed in coordinate system S.

### Transformation S → E

Describes mounting of laser scanner on the robotic end-point. Constants used in following equation could be acquired from documentation of each laser scanner and its mounting holder. It is defined as combination of RPY rotation and linear translation (Solc and Zalud 2006):
3

where *c*(*x*) = *cos*(*x*), *s*(*x*) = *sin*(*x*), *x*_*t*_, *y*_*t*_ and *z*_*t*_ is translation of system *S* in system *E* along appropriate axis, *u*_*t*_ is roll, *v*_*t*_ is pitch and *w*_*t*_ is yaw of system *S* in coordinate system *E*. This transformation is time-invariant and belongs to each scanner. Describes, how scanner is rotated and translated according to the robotic end-point.

### Transformation E → M

Defines transformation between robotic end-point coordinate system (*E*) and robotic manipulator coordinate system (*M*). Parameters in following equation describe actual position of manipulator’s end-point in manipulator’s own coordinate system. It is defined using RPY rotation and translation (Solc and Zalud 2006):
4

where *c*(*x*) = *cos*(*x*) and *s*(*x*) = *sin*(*x*), *x*, *y* and *z* is actual position of manipulator’s end-point in own manipulator’s coordinate system *M*, *u* is its roll, *v* is pitch and *w* is yaw. Parameters used in this transformation are time-variant and could be acquired from manipulators driver.

### Transformation M → 0

Describes relation between coordinate system of manipulator and coordinate system, in which we want to publish results of our 3D scanning. In our case, system 0 is identical with system *M*. If it is not, the homogeneous transformation from system 0 to *M* would be described by matrix *H*_0*M*_ similar to *H*_*ME*_.

Each point measured by laser scanner is transformed using equation 1. Result of these transformations is cloud of points in three-dimensional Cartesian space. These points are then further processed.

## Generation of 3D models

Pure point-cloud visualized in 3D space is not illustrative enough, so our software package of Robotic 3D Scanner contains module, which produces shaded, surface-covered 3D model. This module uses Delaunay Triangulation (Chen 2011) method in order to find three closest points to be connected into the triangle used for rendering image by graphic device (Grootjans 2009).

**Figure 4:**
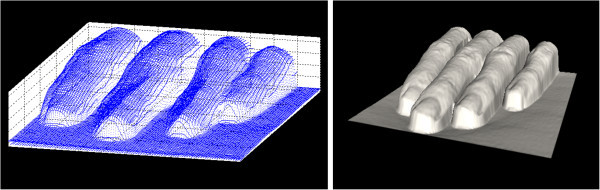
**Measured data visualized as point-cloud and as surface-covered shaded model.** Pure point-cloud figured by dot per point is shown on left side. On right side, there is a surface-covered model,which is much more illustrative.

On Figure 4, comparison of both visualizing modes is displayed.

### Controlling robotic 3D scanner

**Figure 5:**
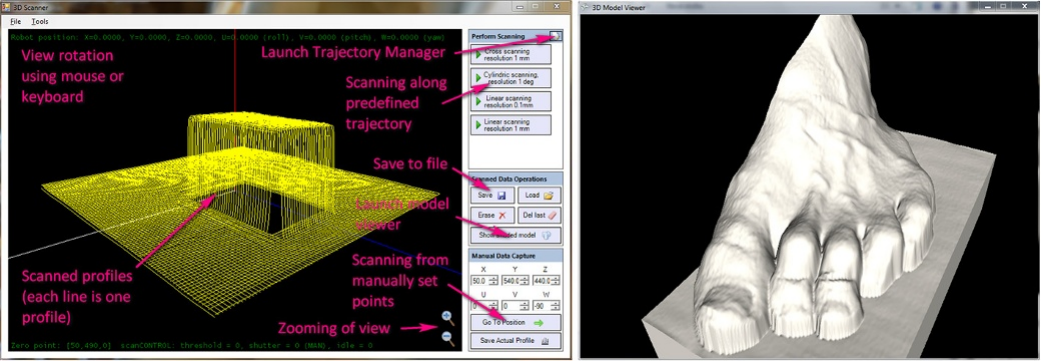
**Graphic User Interface for Robotic 3D Scanner controlling.** Interface for scanner controlling, allowing capturing scans on one click (left). Each point-cloud can be visualized as shaded surface-covered model (right).

Interface is designed to be intuitive as much as possible and provides automatic scanning along predefined trajectories, which can be handled also by inexperienced user (Figure 5). Model of patient body is given easily on one click. For expert users, there are also a lot of settings available, including environment for definition of new scanning trajectories. This empowers a flexibility in use of this scanner.

### Robotic 3D scanner accuracy

Overall accuracy of this imaging system can be defined as maximal distance between computed (measured) value of point coordinates relative to true value of point coordinates at 99.7% of measurements (±3*σ*) (International Vocabulary of Metrology - Basic and General Concepts and Associated Terms (VIM) 2012). This value depends on robotic manipulator accuracy, laser scanner accuracy and precision of holder connecting scanner and manipulator’s end-point. Because this solution is generic and doesn’t depend on actually used devices, also overall accuracy can be generally declared as:
5

where Δ_*M*_ is placing accuracy of manipulator’s end-point, Δ_*S*_ is measuring accuracy of laser scanner and Δ_*H*_ is error in coordinate computation caused by precision of mounting holder.

Last parameter is hardly computable, because this error depends also on actual distance of laser scanner from object, etc. However, this error is systematic, so it is minimizable by calibration, which is described in following section. If we consider calibrated device, overall accuracy is then given by equation:
6

where both Δ_*M*_ and Δ_*S*_ are usually specified in datasheet of manipulator and laser scanner. Δ_*C*_ is a residual error of Δ_*H*_ after calibration, which is discussed in following section and generally Δ_*C*_ ≪ Δ_*H*_. In the worst case, value of Δ_*C*_ could be defined as in equation 8, so the overall accuracy of device according to (International Vocabulary of Metrology - Basic and General Concepts and Associated Terms (VIM) 2012) is then given by equation:
7

Note, that this error estimation is very pessimistic and defines the worst case (3*σ*). In real operation, overall accuracy is usually better.

In our research, we use robotic manipulator Epson C3 with accuracy of end-point placing ±0.02 *mm* (Epson C3 Compact 6-Axis Robot Manual 2011), laser scanner MicroEpsilon ScanCONTROL2750-100 with accuracy ±0.04 *mm* (Instruction Manual scanCONTROL 2008) and laser scanner SICK LMS with accuracy ±4 *mm* (LMS 400 Laser Measurement System Operating Instructions 2006). For capturing human body, we use MicroEpsilon scanner and according to equation 7, overall accuracy in this case is ±0.18 *mm*.

**Figure 6:**
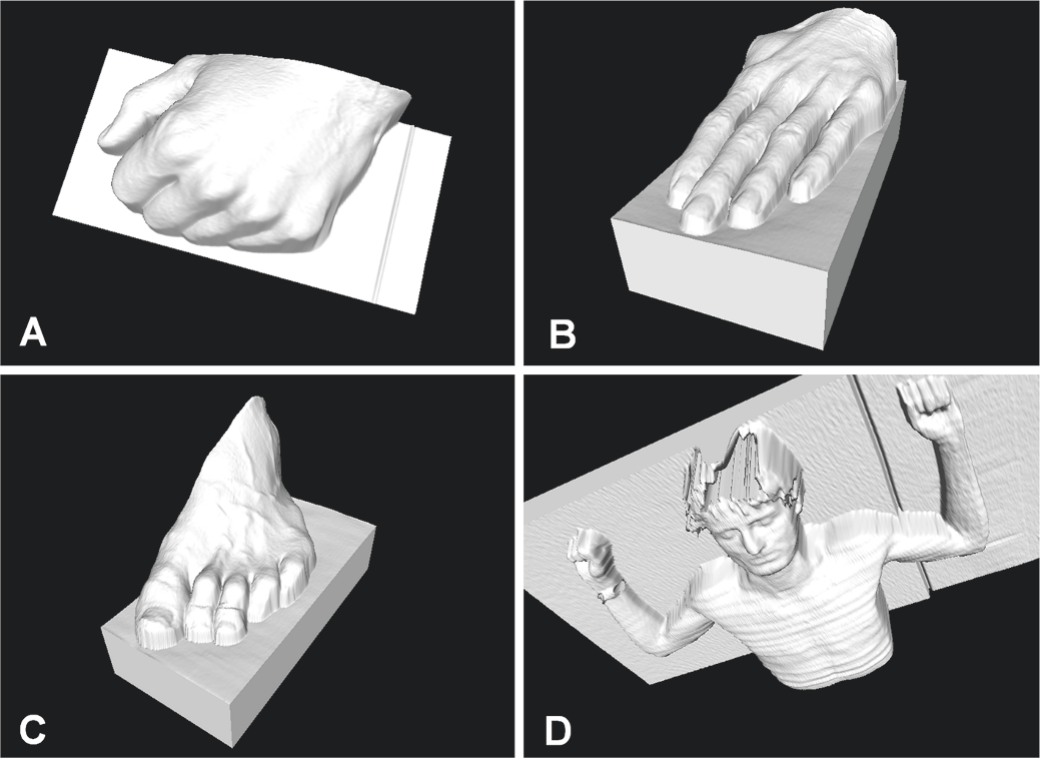
**Examples of human body captured by Robotic 3D Scanner.** Figures **A-C** shows examples of models visualizing parts of human body. These models were captured with precise triangulation laser scanner. Model D was captured for comparison with wide-measuring-range scanner working on TOF principle. When on Fig. **A-C** particular pleats of skins on fingers are visible, figure **D** shows human body more roughly.

Example of models captured with this configuration are on Figure 6A-C. This laser scanner disposes with measuring range only 10 *cm* long (Instruction Manual scanCONTROL 2008), so we can capture just in proximity of manipulator’s operating area. In case of capturing larger structures (e.g. as buildings), we use laser scanner SICK LMS400. In this configuration, overall accuracy is lower (±12.06 *mm*), but measuring range is up to 3 *m* from robotic end-point. Example of model captured with this configuration is on Figure 6D. This configuration is not suitable for scanning human body, it is just for comparison here.

### Scanner calibration

The aim of calibration procedure is to compensate deviations of real dimensions of mounting holder compare to dimensions stated in its documentation. If these deviations are compensated, then equation for overall accuracy (eq. 6) is valid. In fact, it is looking for 6 calibration constants determining translation and rotation of scanner in 6-DOF caused by imprecision of mounting holder.

Proposed method is based on principle, that if some scene is firstly captured from one direction, and then the same scene is captured from another direction, deviations in mounting holder dimensions causes shifting and deforming of scene, but in both cases differently. From differences among these two images of the same scene, we can compute desired calibration constants. This approach would work in case of any scene, but for the simplification of image processing, we use scene of cuboid laying on the flat ground (Chromy 2013).

Significant advantage of this method is, that it is based on general object, without any requirements on its precision. If we use common calibration method based on reference object with known dimensions, it will be very difficult to manufacture reference object with enough precision.

Detailed description of computing of each calibration parameter can be found at (Chromy 2013), here it is not presented in order to be concise.

### Residual error after calibration

Calibration procedure search for identical points in two scans of the same scene and evaluates such transformation, which merges these points together. Lets imagine the worst case of this – there is only one point measured from 2 places. Each representation of point is measured with maximal deviation from true value Δ = Δ_*M*_ + Δ_*S*_, so if we merge there points together, we can cause residual error Δ_*C*_, which maximal value is:
8

Each uncertainty of Δ_*C*_ is composed from uncertainty type A and type B (Palencar et al. 2001). When number of measurements increases, the uncertainty type A is being minimized and measured value converges in direction to true value. Because calibration procedure uses several thousands of points, also residual error Δ_*C*_ after calibration will be smaller than at equation 8.

As a result of this, the overall accuracy will be usually better than maximal guaranteed error defined in equation 7, what is a limit value of error.

### Evaluating benefits of calibration

To be able to evaluate benefits of calibration, the criterion function has been introduced. For each point from one scan, distance to every point from second scan is computed and square of distance to the nearest point is added to the sum:
9

where *I*_1_ and *I*_2_ are scans which correlation we are looking for and ∥*xy*∥ is a distance between points x and y. The task of calibration procedure is to compute such combination of calibration constants, which provides the smallest value of *f*_*err*_.

Beside the criterion function values, also average-absolute error Δ_*abs*_ and root-mean-square error Δ_*abs*_ were computed in order to provide illustrative demonstration of calibration influence using equations (Lehmann and Casella 1998):
1011

**Figure 7:**
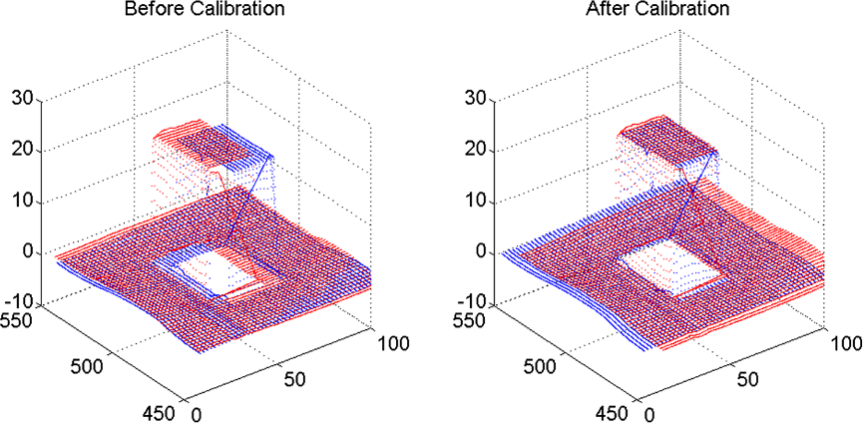
**Influence of calibration.** On the left, point-cloud measured without previous calibration is presented. On the right, there is a point-cloud measured by calibrated device. On both figured, two scans of one scene captured from different positions are presented. One scan is displayed in blue, second in red.

Influence of calibration is demonstrated on Figure 7 and in Table 1.

**Table 1:** **Illustrating influence of calibration**

	Before calibration	After calibration
Criterion function *f*_*err*_ [–]	60 215.61	76.41
Average-absolute error Δ_*abs*_ [mm]	2.907	0.085
Root-mean-square error Δ_*RMS*_ [mm]	4.044	0.144

Values of criterion function demonstrates benefits of proposed calibration procedure.

## Comparison with standard modalities

Parameters, advantages and disadvantages of common actually used imaging modalities, which are used in case, when patient body surface capturing is required, are summarized in Table 2. Proposed Robotic 3D Scanner is compared with them in order to analyze benefits of its use.

**Table 2:** **Comparison of various medical imaging modalities**

	Sonograph	CT	MRI	Robotic 3D
	(Musil et al. 2008 ),	(MCCollough and Zink 1999 ),	(Novelline 1997 ),	
	(Compare Ultrasound Cost 2012 ),	(Herman and Gabor 2010 ),	(Magnetic Resonance Imaging 2010 )	
	(Physics of Ultrasound Imaging 2009 )	(Statni urad pro jadernou bezpecnost 2002 )		
		(Compare CAT Scan Cost 2012 )		
Acquisition costs	150–750 EUR	120 000 EUR	775 000 EUR	38 000 EUR
Price per scan	< 1 EUR	100–250 EUR	230–410 EUR	< 1 EUR
Scanning time	< 1 min.	3–10 min.	20–40 min.	1–2 min.
Resolution	10–50 mm	up to 0.2 mm	Up to 0.5 mm	< 0.18 mm
Harmful radiation	No	Yes	No	No
Limitations	None	Must be use rarely	No metal objects	none
Output	Inner structures	Inner structures	Inner structures	Body surface

Illustrates benefits of use of Robotic 3D Scanner in cases, where patient body surface 3D model is sufficient.

Sonographs are fast and relatively cheap devices, but do not meet required minimal resolution. Also commonly used device are intended to watch just inner structures and devices capable of scanning surface of body are very rare (Physics of Ultrasound Imaging 2009).

Computed tomography reaches desired resolution, but it also cannot be used because of high expositions of ionizing radiation (Statni urad pro jadernou bezpecnost 2002). Repeated measurements cannot be performed because of this reason. CT devices are also very expensive at acquisition and also at operation.

The only device, which is useful for our purposes is MRI. It disposes with sufficient resolution and does not produce harmful radiation, so it can be used repeatedly. But its acquisition costs are extremely high, as well as its operational costs (Magnetic Resonance Imaging 2010). It is one of the most expensive medical imaging, so as a result of this, not enough devices are available. It leads to long waiting times for examination, what makes problems when periodical scanning is required. Problems are also with people afflicted with claustrophobia, with pacemakers, endoprostheses or with metal piercing.

All these disadvantages are not present at Robotic 3D Scanner. Its clear, that it cannot supply MRI completely – MRI is designed mainly for models of interior of body. But in many cases, where look into the body is not necessary, MRI can be successfully replaced by Robotic 3D Scanner. In such cases, its use saves considerable amounts of money, scanning is faster and we don’t block patients, that must be captured by MRI and cannot be scanned with other device. Also the comfort of patient is high, because of no special requirements on him/her.

## Conclusions

This paper describes Robotic 3D Scanner as an alternative to Magnetic Resonance Imaging in special cases, where information required for examination is not the whole 3D model of afflicted part of body, but only its surface. In these cases, Robotic 3D Scanner, a device specially developed for these situations, can provide at least the same quality of resulting 3D scan in faster time and with lower expenses. In most cases, the result reaches the higher resolution than MRI and brings higher comfort for patient.

The prototype of Robotic 3D Scanner has been developed, which is fully capable of building 3D scans. In future work, the safety functions will be more developed in order to be sure, that operation of manipulator cannot be harmful for its users and scanned patients at any circumstances. Scanning capability will be also extended with multi-spectral texture mapping, that shows information of colours and temperatures on the surface of 3D model.

Entire research contributes to optimization of public health care operation by avoiding expensive examinations, which could be performed in cheaper and smarter way. This research was performed on behalf of St. Anne’s University Hospital Brno, where application of proposed Robotic 3D Scanner is planned.

### Ethical approval

All the experiments were perfomed in Compliance with the Helsinki Declaration and persons taking part in it gave their approval to perform the experiment.
